# Common ground: The foundation of interdisciplinary research on bat disease emergence

**DOI:** 10.1371/journal.pbio.3000947

**Published:** 2020-11-09

**Authors:** Rebekah C. Kading, Tigga Kingston

**Affiliations:** 1 Department of Microbiology, Immunology, and Pathology, Colorado State University, Fort Collins, Colorado, United States of America; 2 Department of Biological Sciences, Texas Tech University, Lubbock, Texas, United States of America

## Abstract

Human perturbation of natural systems is accelerating the emergence of infectious diseases, mandating integration of disease and ecological research. Bats have been associated with recent zoonoses, but our bibliometric analysis of coauthor relationships identified a separation of bat ecologists and infectious disease researchers with few cross-disciplinary relationships. Of 5,645 papers, true interdisciplinary collaborations occurred primarily in research focused on White Nose Syndrome (WNS). This finding is important because it illustrates how research with outcomes favoring both bat conservation and disease mitigation promotes domain integration and network connectivity. We advocate for increased engagement between ecology and infectious researchers to address such common causes and suggest that efforts focus on leveraging existing activities, building interdisciplinary projects, and networking individuals and networks to integrate domains and coordinate resources. We provide specific opportunities for pursuing these strategies through the Bat One Health Research Network (BOHRN).

## Abstract 2

Limited collaboration between bat ecologists and disease researchers has precluded integrative research on disease emergence. These historical silos are now challenged by the emergence of SARS-CoV-2, which threatens both human and bat health. This Perspective article maintains that interdisciplinary research can be accelerated when disparate domains address common, foundational causes.

Months into the COVID-19 pandemic, the pathway to its zoonotic emergence has yet to be characterized. This is not unusual; understanding disease emergence requires integration of expertise from diverse domains in complex ecological and epidemiological contexts [1]. Although such interdisciplinarity is central to One Health frameworks [2], Manlove and colleagues [3] demonstrated that too often, the requisite expertise is siloed, limiting integrative understanding of complementary fields. Here, we focus specifically on the bat research and conservation communities, in which historical silos are now challenged by the emergence of SARS-CoV-2. Similar divisions likely exist in research communities focused on other taxa that harbor zoonotic pathogens, such as rodents (hantaviruses and arenaviruses), birds (influenza viruses), nonhuman primates (retroviruses), and wild ungulates (prions).

With more than 1,420 known species, bats are a critical (yet highly vulnerable) component of ecosystems worldwide [4]. Some bat species are also the reservoir hosts of zoonoses that have severe human health consequences (rabies, Nipah, Marburg) and others have been implicated in the emergence of the human pathogenic coronaviruses SARS, Middle East Respiratory Syndrome (MERS), and now SARS-CoV-2 [5]. This duality has led to entrenched positions within the bat and virus research communities, with accusations of alarmist risk inflation to support funding of viral discovery on the one hand and denial or down-playing of the role of bats in emerging infectious diseases on the other. Media representation has further polarized positions, with emotive headlines that refer to bats as, for example, “breeding grounds of deadly diseases” (https://www.discovermagazine.com/health/why-bats-are-breeding-grounds-for-deadly-diseases-like-ebola-and-sars) or “the number-one carrier of disease” (https://time.com/4827511/bats-viruses-diseases-pandemic/).

The consequences of the COVID-19 pandemic for human health are apparent, but negative outcomes for bat populations and conservation are also concerning. Bat conservation has always been challenged by public perceptions of bats, but the misperception that bats carry the circulating pandemic strain of SARS-CoV-2 has led to lethal persecution [6], evictions from roosts, and has compromised existing conservation programs [7]. Moreover, there is a small but credible risk that SARS-CoV-2 could be transmitted from humans to bats, with unknown consequences for bat health [8]. Thus, SARS-CoV-2 threatens both human and bat health, but advancing the missions of bat conservation and public health protection has seemed, to many, to conflict, hampering the integrative research needed to characterize and mitigate disease emergence.

To investigate cross-disciplinary collaboration between ecological- and infectious disease-oriented bat researchers, we undertook a bibliometric analysis of coauthor relationships. Publication metadata were extracted from the Web of Science database on over 5,600 journal articles published between 1950 and 2019 (see S1 Text). Consistent with the divisions observed between ecology, veterinary, and other professionals working in a broader One Health context [3], our analysis revealed a clear boundary between authors representing disease- and ecology-focused disciplines in bat research (Fig 1) and distinct clusters within disciplines. Discipline-specific expertise is the bedrock of collaborative research, so disciplinary clusters of productive research groups are expected and needed [9]. Eighteen highly connected, influential authors were identified (betweenness centrality >500), but only nine published with colleagues outside their primary discipline (Fig 1: Authors “A” from disease and “B” from ecology). Moreover, five of these nine “boundary-crossing” authors came from the same cluster containing author “B” (Fig 1). Other influential authors, while extraordinarily productive within their own fields, have not published outside of their discipline (Fig 1: Authors “C” in disease and “D” in ecology).

**Fig 1:**
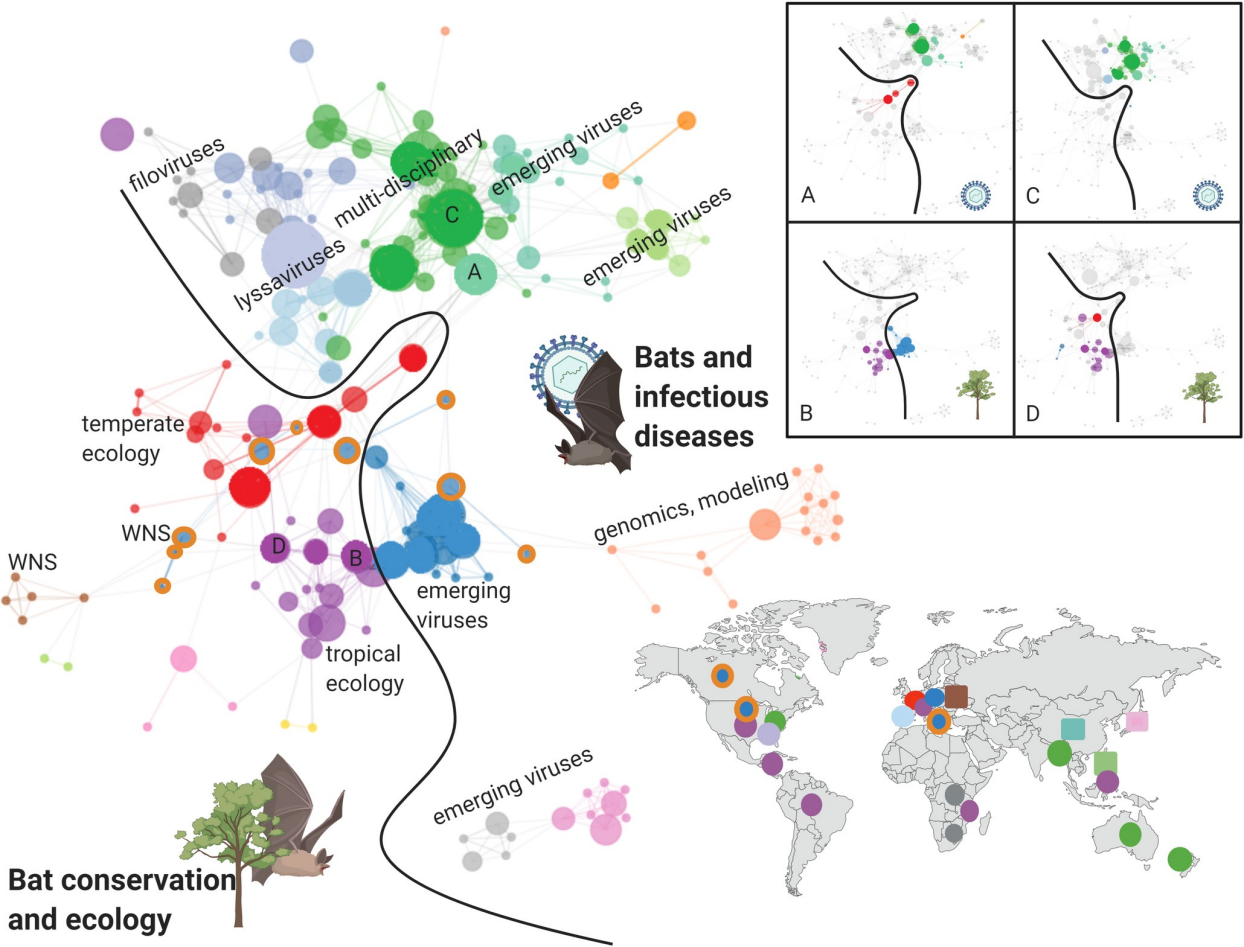
Coauthorship network of the 200 most-published bat researchers between 1950 and 2019. Map shows location of institutional affiliations of authors in each cluster. Colors correspond to the author network clusters; squares denote apparent segregation of research groups geographically in addition to topic area. Inset shows the publication networks of four influential authors with betweenness centrality scores >500. Authors "A" and "B" are boundary-crossing authors with collaboration networks that span clusters in both ecology and disease topic areas. Authors "C" and "D" are widely connected within either the ecology or disease communities but do not collaborate across disciplines. Clustering was also driven by geographical and institutional boundaries associated with programmatic missions or funding (inset map) promoting homogenous perspectives within the cluster and potentially retarding dissemination of findings. See S1 Text for additional detail on this analysis. *This figure was created with permission using BioRender*.*com*. WNS, White Nose Syndrome.

So how can we increase connectivity and strengthen the network? Our analysis highlighted the power of a common goal to overcome institutional, cultural and trust barriers and accelerate interdisciplinary collaboration [2,9]. This was exemplified by an international cluster focused on White Nose Syndrome (WNS) (blue/orange in Fig 1), a disease resulting from a fungal pathogen that has killed millions of bats in North America since it was first detected in 2006. The WNS cluster repeatedly crosses disciplinary lines in the network, and its diverse membership produced interdisciplinary papers that combined principles of ecology with those of infectious disease to address themes relevant to the study of disease emergence in any pathogen system, e.g., host/pathogen interactions, pathogen transmission in community networks, disease effects on macroecological patterns of bats, invasion ecology, seasonal transmission dynamics, and transcriptomics (see S1 Data).

Just as WNS provides common ground for convergent research, understanding and mitigating other emerging zoonoses with One Health implications, like SARS-CoV-2, involve common challenges that are best met through cross-disciplinary engagement. This engagement can span robust data collection for a complementary discipline (**leveraging**), interdisciplinary projects designed collaboratively from the ground up (**building**), and research networks that actively aim to integrate domains and resources (**networking**):

1. **Leveraging** existing research programs can further common agendas. For example, characterizing the distribution of bat diversity and resolving taxonomic questions is both central to effective bat conservation [10] and needed to draw correct associations between pathogens and hosts. Many viral discovery/surveillance papers do not identify bat hosts to the species level, in part because bat diversity of many regions of biosurveillance interest is poorly known, with unresolved taxonomy of species-rich groups, cryptic species complexes, and likely many undescribed species. Surveillance teams can lever their programs to contribute and benefit by doing the following: i) integrating biodiversity expertise (ecologists, taxonomists, etc.) and infrastructure into the team from the outset [11] (this would facilitate species identifications and robust integration of ecological principles into study design); ii) sequencing tissues collected for pathogen detection for host species markers and contributing to reference databases (currently, > 800 bat species are represented in the Barcode of Life Database [BOLD] of DNA sequences matched to identified voucher specimens); and iii) depositing voucher specimens with curated collections (prioritizing in-country national or university collections). Preservation of the cold chain in surveillance work presents an unparalleled opportunity for disease teams to contribute high-quality (flash-frozen) material to the global effort to sequence the full genome of all living bat species (Bat 1K— https://bat1k.ucd.ie/), an initiative that is already paying dividends for understanding tolerance to viral infection in bats [12].
2. **Building** new integrative research areas that are foundational to both research domains provides strong motivation for collaboration. The question “What is a sick bat?” is central to global discussion of the ability of bats to harbor infectious agents that are highly pathogenic to people but with (usually) little apparent health impact to themselves (reviewed by [5]). Understanding bat health may provide important biomedical insights regarding infection tolerance. Bat health is also a key concept in studies of anthropogenic and environmental stressors that both threaten species conservation [4] and influence pathogen shedding and infection dynamics [1,13]. Similarly, human–bat interactions, such as bat hunting and consumption, are relevant to both bat conservation and cross-species transmission of pathogens and would benefit from greater integrative effort to characterize the intersection of bat biology and ecology (e.g., bat movement, reproductive phenology, and resource availability), drivers of human behavior (e.g., economics, seasonality, attitudes, and social norms) and disease dynamics. There is extensive guidance on the development of interdisciplinary teams and research in the literature [2,9], but the critical first step is for experts in different domains to network and build professional relationships based on mutual understanding and trust [2].
3. **Networking** individuals and existing networks builds and reinforces relationships that accelerate the transfer of knowledge and expertise and allow for coordination of activities and key resources [14]. Many ecologists within our coauthor network have access to long-term study sites or wild study populations, key resources with years and sometimes decades of relevant data on ecology, life history, genetic relationships, responses to disturbance regimes, etc. These established sites and populations could provide settings for pathogen studies across ecological and conservation contexts. Conversely, numerous scientific questions can only be rigorously addressed with the use of captive bat colonies. Such colonies are few but distributed across the coauthor network in support of research that ranges from the biomechanics of flight and the evolution of sociality to experimental challenges with infectious agents to determine susceptibility and disease dynamics relevant to human or bat health. Ideally, networking of existing colonies could facilitate access to representatives from each bat family, comparison of biological phenomena across taxa, and generation of associated primary cell lines, genomic data, and transcriptomic data. While there is clear potential for colonies held for nondisease research to make noninvasive or minimally invasive contributions to a network of colonies, this can be a contentious issue that is most likely to be advanced in a network that has established trust and respect among members.

We invite readers to join the Bat One Health Research Network (BOHRN) (https://www.bohrn.net/), an initiative established in 2017 to bring together disease and bat researchers to characterize global threats of bat-borne pathogens in a conservation-conscious One Health framework. Efforts focus on the following: host–pathogen interactions; pathogen surveillance, diagnostics, and epidemiology; bat ecology in natural and human-modified landscapes; and human–bat interactions. BOHRN members include ecologists, conservation biologists, immunologists, virologists, veterinary scientists, and modelers. To facilitate network building and enable researchers to connect with complementary experts, members have access to a membership directory and receive notification of BOHRN events, funding opportunities, conferences receptive to interdisciplinary or cross-disciplinary presentations, and symposia. The diverse global membership is committed to the development of more transparent, open, and collaborative research and practices and is invited to contribute to or join the working groups addressing the thematic foci that establish our common ground.

## Supporting information

S1 Text Web of Science search parameters and bibliometric analysis methods. (DOCX)

S1 Data Full data set used in the bibliometric analysis. Highlighted fields represent a few selected publications that integrate ecological and infectious disease concepts.
(XLSX)

## Abbreviations

BOHRN: Bat One Health Research Network
BOLD: Barcode of Life Database
BTRP: Biological Threat Reduction Program
DTRA: Defense Threat Reduction Agency
MERS: Middle East Respiratory Syndrome
WNS: White Nose Syndrome
