# Supplementary material for: Common ground: The foundation of interdisciplinary research on bat disease emergence
Source: PLoS Biol. 2020 Nov 9;18(11):e3000947. doi: 10.1371/journal.pbio.3000947 (PMC7676706; doi:10.1371/journal.pbio.3000947)
Supplement: S1 Text — (DOCX) [file pbio.3000947.s001.docx]

**Supplementary Methods**

From ISI Web of Science (WoS), we extracted papers under the topic “bats” or “chiroptera” from 1950 – 2019. The resulting 28,001 citations were refined to 7,425 by filtering for articles indexed by SCI and SSCI in the WoS categories “ecology”, “multidisciplinary sciences”, “biodiversity conservation”, “virology”, “infectious diseases”, and “immunology”. Unrelated papers were manually removed, reducing the final dataset to 5,645 papers. Records were imported into Bibliometrix (1). We employed co-author analysis to analyze the social structure of the field (1) and build a network map of authors (nodes) linked by co-authorships (Fig 1). Clusters, generated with the Walktrap algorithm, comprised authors who published together significantly more frequently than with others. Primary research themes of clusters were identified from professional experience and inspection of publications. Betweenness centrality scores for each author (range: 0 - 2,250) were calculated (2). Authors with high betweenness centrality connect different parts of the network, either within or across disciplinary boundaries.

1. Aria M, Cuccurullo C. Bibliometrix: An R-tool for comprehensive science mapping analysis. J Informetr. 2017;11: 959–975.

2. Pierce SJ. Boundary crossing in research literatures as a means of interdisciplinary information transfer. J Am Soc Inf Sci. 1999;50: 271–279.
